# Reduced Circulating Levels of miR-433 and miR-133b Are Potential Biomarkers for Parkinson’s Disease

**DOI:** 10.3389/fncel.2017.00170

**Published:** 2017-06-23

**Authors:** Xiong Zhang, Rui Yang, Bei-Lei Hu, Pengcheng Lu, Li-Li Zhou, Zhi-Yong He, Hong-Mei Wu, Jian-Hong Zhu

**Affiliations:** ^1^Department of Geriatrics and Neurology, the Second Affiliated Hospital and Yuying Children’s Hospital, Wenzhou Medical University, WenzhouChina; ^2^Department of Preventive Medicine, Wenzhou Medical University, WenzhouChina; ^3^Department of Biostatistics Graduate Program, University of Kansas Medical Center, Kansas CityKS, United States; ^4^Key Laboratory of Watershed Science and Health of Zhejiang Province, Wenzhou Medical University, WenzhouChina

**Keywords:** Parkinson’s disease, plasma, microRNA, biomarker, miR-433, miR-133b

## Abstract

Aberrant expression of microRNA (miRNA) in tissues may lead to altered level in circulation. Considerable evidence has suggested that miRNA deregulation is involved in the pathogenesis of Parkinson’s disease (PD). In this study, we screened a set of PD-associated miRNAs and aimed to identify differentially expressed miRNAs in plasma of PD patients and to evaluate their potentiality to serve as PD biomarkers. A total of 95 subjects consisting of 46 sporadic PD cases and 49 controls were recruited. Plasma levels of six miRNAs including miR-433, miR-133b, miR-34b, miR-34c, miR-153, and miR-7 were evaluated using reverse transcribed quantitative PCR, among which we found that miR-34c and miR-7 were below detection limit under our condition. The results showed that levels of circulating miR-433 (*P* = 0.003) and miR-133b (*P* = 0.006), but not miR-34b and miR-153, were reduced in PD patients. miR-433 and miR-133b were strongly correlated in both control and PD groups (*r*_s_ = 0.87 and 0.85, respectively). The correlation between miR-34b and miR-153 expressions was significantly reduced (*P* < 0.05) in the PD group. Although miR-433 and miR-133b were likely to be functionally complimentary as suggested by Pathway and Gene Ontology analyses, these two miRNAs *per se* might not be sufficient to predict PD. No correlation was observed between the four miRNAs and age or severity of disease. Collectively, our results demonstrate that circulating miR-433 and miR-133b are significantly altered in PD and may serve as PD biomarkers.

## Introduction

Parkinson’s disease (PD) is the second most common neurodegenerative disease characterized by progressive loss of dopaminergic neurons in substantia nigra. While hereditary PD is mainly caused by gene mutations such as in *SNCA, Parkin*, and *PINK1*, the majority of this disease is sporadic and the pathogenesis involves both genetic vulnerabilities and environmental exposures (Bekris et al., 2010). Thus far, diagnosis of PD largely relies on the presence of motor symptoms, clinically characterized by bradykinesia, resting tremor, rigidity, and at late stage gait instability. However, the actual onset of this disease precedes the motor manifestations by a number of years (Kalia and Lang, 2015). As a consequence, possible clinic or preventive interventions lag far behind the disease onset. Substantial efforts have therefore been made to develop potential markers for early diagnosis of PD, and the strategies include prodromal clinical signs, imaging, skin or colonic biopsies, genetic sequencing, and biochemical testing in cerebral spinal fluid, blood, saliva, and urine. For example, certain non-motor symptoms such as olfactory impairment and rapid eye movement sleep behavior disorder, which are frequently present in PD before motor manifestations, have been under investigation as potential markers for PD diagnosis (Postuma et al., 2012). Unfortunately, thus far none of them has been proven effective in clinical trials despite early promises (Kalia and Lang, 2015).

MicroRNAs (miRNAs) act as endogenous regulators of gene expression by binding to complementary sequences of target messenger RNA (Chen and Rajewsky, 2007). Being small non-protein coding RNAs and also relatively stable, disturbance of miRNA in tissues is possibly reflected by changes in serum/plasma, which renders miRNA a potentially convenient biomarker in monitoring diseases (Reid et al., 2011). miRNAs have also been suggested to play an important role in PD and profiled in brains of PD patients (Kim et al., 2007; Minones-Moyano et al., 2011). Among the miRNAs known to be associated with PD, in particular those with postmortem evidence and/or regulating α-synuclein expression, are: miR-34b and miR-34c, down-regulated in the brains of PD patients (Minones-Moyano et al., 2011; Villar-Menendez et al., 2014) and repressing α-synuclein expression (Kabaria et al., 2015); miR-7 and miR-153, suppressing α-synuclein expression (Junn et al., 2009; Doxakis, 2010); miR-433, regulating fibroblast growth factor 20 (FGF20) levels and subsequent α-synuclein expression (Wang et al., 2008); and miR-133b, down-regulated in midbrain of PD patients and interacting with Pitx3 (Kim et al., 2007). In this study, we focused on these six miRNAs to better understand PD-associated changes and explore their potentiality as PD biomarkers.

## Materials and Methods

### Study Subjects

A total of 95 Han Chinese individuals were enrolled in this study, consisting of 46 sporadic PD patients (22 men and 24 women) and 49 controls (22 men and 27 women). The age of the patients and controls was 63.13 ± 1.46 and 60.35 ± 1.16, respectively. The two groups were comparable by gender (*P* = 0.78) and age (*P* = 0.14). The patients were diagnosed by two movement disorder neurologists according to the UK Parkinson’s Disease Society Brain Bank Criteria, and were not with a family history of PD or secondary and atypical parkinsonism. The control subjects were free of neurological disorders by medical history, physical and laboratory examinations. The PD patients were divided into three subgroups according to Hoehn and Yahr (H&Y) staging (Hoehn and Yahr, 1998) including stage I (H&Y stage: 1–2), stage II (H&Y stage: 3–4), and stage III (H&Y stage: 5). All subjects participating in this study signed informed written consents. The study was approved by the ethics committee of the Second Affiliated Hospital and Yuying Children’s Hospital, Wenzhou Medical University.

### Plasma Sample Collection and miRNA Extraction

Fasting blood samples were collected in EDTA tubes and centrifuged for 15 min at 1500 rpm at room temperature. After centrifugation, plasma was collected and stored at -80°C until analysis. RNAs enriched of small size were extracted from 500 μl of aliquots using miRcute miRNA isolation Kit (Tiangen, Beijing, China) according to the manufacturer’s instruction.

### Determination of miRNA Levels by RT-qPCR

Sequences and miRBase accession numbers of the assessed miRNAs were listed in **Table 1**. RNA (50 ng) was reverse-transcribed using FastQuant RT Kit with gDNase (Tiangen, Beijing, China). qPCR was performed using FastStart Essential DNA Green Master (Roche, Mannheim, Germany) in accordance with the manufacturer’s instruction in CFX Connect^TM^ Real-Time PCR detection system (Bio-Rad Laboratories, Hercules, CA, United States). Small nucleolar RNA U6 was used as an internal control. The RT and PCR primers for each miRNA and U6 were purchased from RiboBio (Guangzhou, China), termed Bulge-Loop^TM^ miRNA RT-qPCR (reverse-transcription quantitative PCR) primer set and U6 stem-loop RT-qPCR primer set, respectively. Each reaction was performed with triplicates with cycling conditions as follows: 95°C for 10 min, followed by 45 cycles of 95°C for 10 s, 60°C for 15 s, and 72°C for 20 s. The miRNA expression levels were calculated as 2^-Δct^, wherein the average cycle threshold (McCulloch et al., 2008) value of each miRNA was subtracted by their respective Ct value of U6.

**Table 1:** The miRNA sequences and miRBase accession numbers.

Assayed miRNA	Sequence	Accession number
hsa-miR-433-3p	AUCAUGAUGGGCUCCUCGGUGU	MIMAT0001627
hsa-miR-133b	UUUGGUCCCCUUCAACCAGCUA	MIMAT0000770
hsa-miR-34b-3p	CAAUCACUAACUCCACUGCCAU	MIMAT0004676
hsa-miR-34c-5p	AGGCAGUGUAGUUAGCUGAUUGC	MIMAT0000686
hsa-miR-7-5p	UGGAAGACUAGUGAUUUUGUUGU	MIMAT0000252
hsa-miR-153-3p	UUGCAUAGUCACAAAAGUGAUC	MIMAT0000439

### Pathway and Gene Ontology Analyses of miRNA Targets

The miRNA target genes were sequentially analyzed using miRTarBase^1^ (release 4.5; a database that has experimentally validated the miRNA-target interactions; Hsu et al., 2014), and in DAVID^2^ (Database for Annotation, Visualization and Integrated Discovery; Huang et al., 2009), a web-accessible program that integrates functional genomic annotations with intuitive graphical summaries, to identify KEGG (Kyoto Encyclopedia of Genes and Genomes) pathways (Kanehisa and Goto, 2000), followed by gene ontology (GO) analyses (Ashburner et al., 2000; Gene Ontology Consortium, 2004).

### Supervised Learning Algorithms

Six algorithm models were generated using R (version 3.1.2), including decision tree (Quinlan, 1986), conditional inference tree (Hothorn et al., 2006), random forest (Rodriguez et al., 2006), support vector machine (Cortes and Vapnik, 1995), naive Bayesian (Leung, 2007), and logistic regression (Hosmer and Lemeshow, 2000). Model performance was assessed using the area under the receiver operating characteristic curve (AUC), accuracy, sensitivity and specificity. The model metrics were obtained from internal fivefold cross-validation with 100 repeats.

### Statistical Analysis

Statistical analysis was performed using Statistical Product and Service Solutions (SPSS; version 20.0) for windows. Differences in gender and age between patients and controls were assessed using χ^2^ test and *t*-test, respectively. Differences in miRNA expression levels between patients and controls were evaluated using Mann–Whitney *U*-test following normality analysis using Kolmogorov–Smirnov test and Shapiro–Wilk test with or without logarithm and square root transformations. Outliers, which differed more than 30 times from the median, were excluded by using informal box plots to pinpoint the outlying points (Laurikkala et al., 2000). Spearman’s rank correlation coefficient was calculated to estimate the association between levels of each miRNA. Fisher *Z* transformation was used to analyze differences in correlation coefficients of the miRNA pairs between the patient and control groups, and a *z* value outside of the range of -1.96∼1.96 was considered as *P* < 0.05. Values were expressed as means ± SE unless otherwise indicated. A difference was considered statistically significant when a two-tailed *P*-value was <0.05.

## Results

### Expression of the Six miRNAs in Plasma of PD Patients and Controls

**FIGURE 1:**
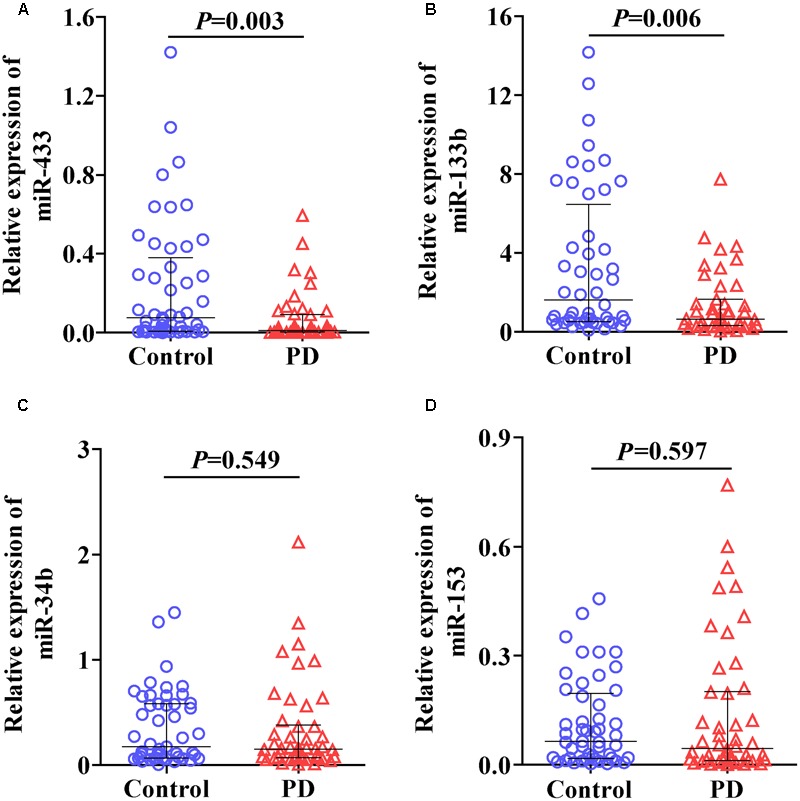
Levels of miR-433 **(A)**, miR-133b **(B)**, miR-34b **(C)**, and miR-153 **(D)** in plasma of Parkinson’s disease (PD) patients and controls. Expression of miRNAs was expressed relative to their respective level of U6. The bar represents median with interquartile range.

We first randomly selected three control and three PD samples to determine whether the six miRNAs could be reliably detected in plasma under our condition as described in Section “Materials and Methods.” An average qPCR Ct value of 40 was considered as a detection limit in this study as values beyond indicate minute abundance (Gallo et al., 2012; Muller et al., 2014). The results showed that levels of miR-34c (Ct, 41.5 ± 0.41) and miR-7 (beyond detection limit) were very low in plasma. In contrast, the other four miRNAs, including miR-433 (Ct, 38.1 ± 1.57), miR-133b (Ct, 33.2 ± 0.29), miR-34b (Ct, 37.5 ± 0.94), and miR-153 (Ct, 38.9 ± 0.65), were constantly detectable. We thus measured and analyzed expression levels of these four miRNAs in the following experiments. The expression levels of miR-433 (**Figure 1A**; *P* = 0.003) and miR-133b (**Figure 1B**; *P* = 0.006) were significantly lower in plasma of PD patients compared with the controls. In contrast, no significant difference was found in miR-34b (**Figure 1C**) and miR-153 (**Figure 1D**) between the controls and PD cases. Of note, four samples (one in controls; three in PD) were excluded in the group of miR-133b based on the outlier calculation. Some studies did not use any controls but kept the reaction system consistent throughout experiments, and straightforwardly used the measured levels (Chen et al., 2008; Ren et al., 2013). The current study followed a consistent system as well; we thus applied this method by removing the normalization of U6, and detected similar results (Supplementary Figure S1).

### Analysis of Correlation between the Four miRNAs in PD Patients and Controls

**FIGURE 2:**
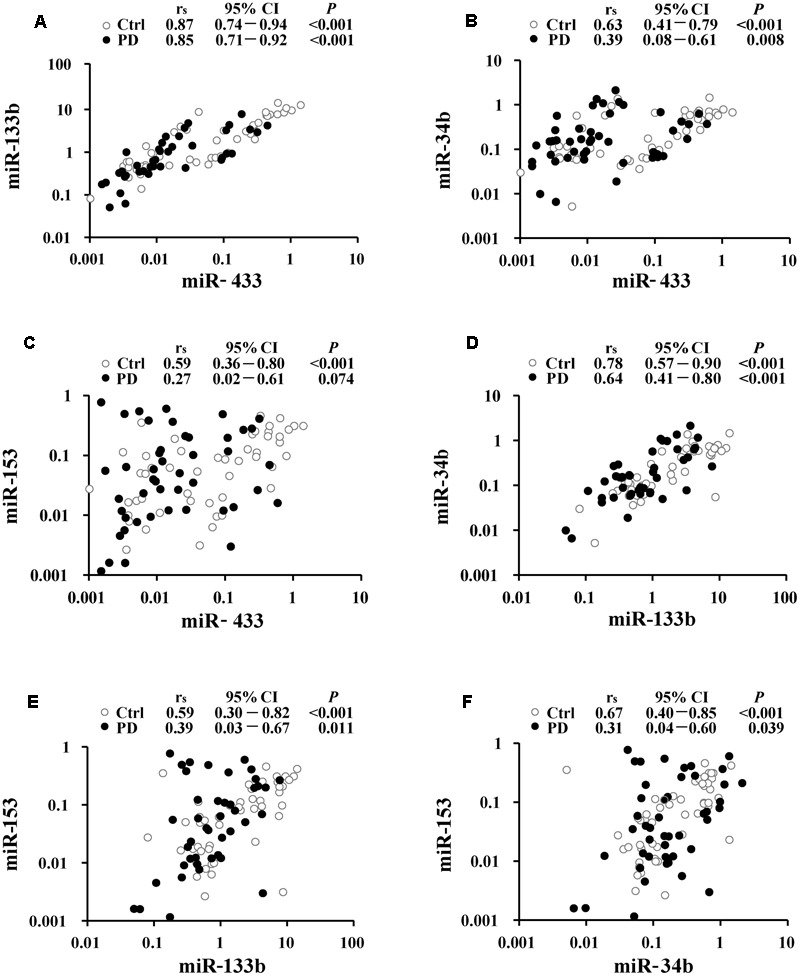
Correlation analyses between miR-433 and miR-133b **(A)**, miR-433 and miR-34b **(B)**, miR-433 and miR-153 **(C)**, miR-133b and miR-34b **(D)**, miR-133b and miR-153 **(E)**, as well as miR-34b and miR-153 **(F)** in Parkinson’s disease (PD) and control (Ctrl) groups. Spearman’s rank correlation coefficient (*r*_s_) along with 95% confidence intervals (CI) and *P*-values are listed above each chart.

Correlations were analyzed to understand potential links within miR-433, miR-133b, miR-34b, and miR-153. The strongest correlation was present between miR-433 and miR-133b (*r*_s_ = 0.87 and 0.85, respectively, in the control and PD groups; **Figure 2A**), followed by the correlation between miR-133b and miR-34b in the controls (*r*_s_ = 0.78; **Figure 2D**). The results showed a pattern of reduction in miRNA correlations from the controls to patients (**Figure 2**). However, only the coefficient of the pair of miR-34b and miR-153 was significantly reduced in PD cases compared with that in the controls (*z* = 2.31; **Figure 2F**). In contrast, the other reductions from the controls to patients were not significant (**Figures 2A–E**; *z* = 0.35 for the pair of miR-433 and miR-133b; *z* = 1.55 for miR-433 and miR-34b; *z* = 1.89 for miR-433 and miR-153; *z* = 1.32 for miR-133b and miR-34b; *z* = 1.22 for miR-133b and miR-153). No correlation was found between age and miRNA expression levels in both control and PD groups (Supplementary Table S1). We also analyzed whether levels of the miRNAs were associated with disease severity, but found no difference among the three stages of our PD patients (Supplementary Table S2).

### Pathway Analysis, GO Analysis, and PD Prediction Modeling

**FIGURE 3:**
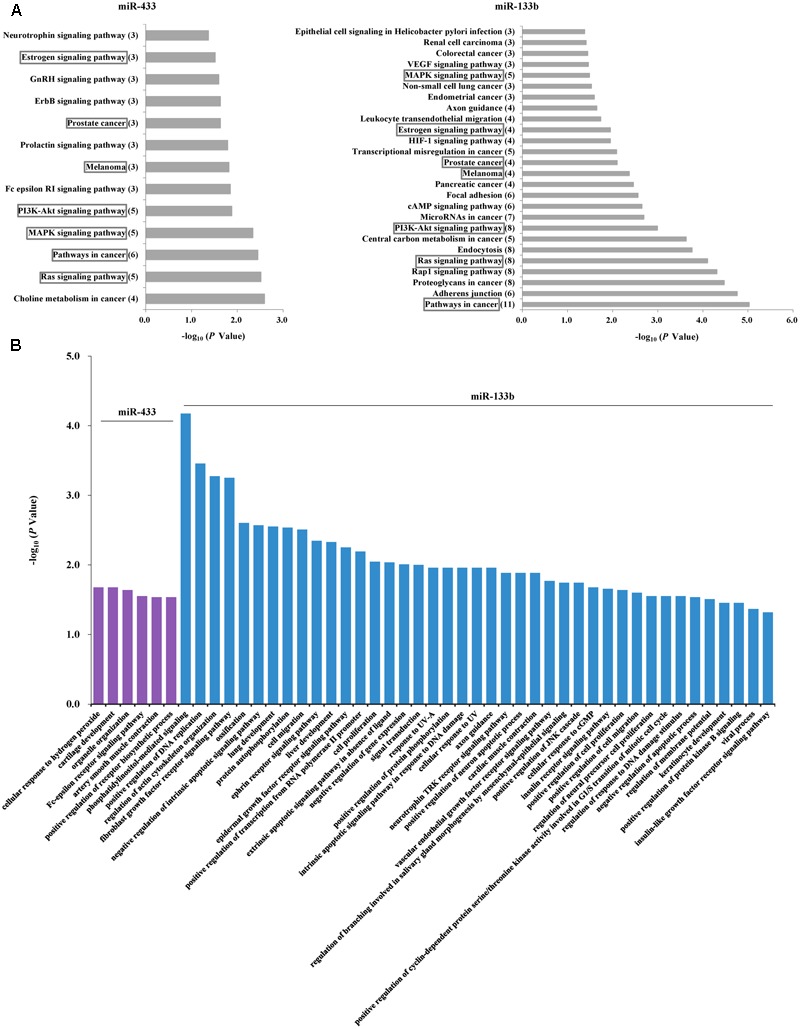
Analyses of potential target genes for miR-433 and miR-133b. **(A)** KEGG pathway analysis of the target genes. The horizontal axis represents enrichment score. The number of enriched genes for each pathway is shown in parentheses in the vertical axis. The boxes indicate the common pathways shared by miR-433 and miR-133b. **(B)** Gene ontology analysis of the target genes. *P*-value was to examine the significance of gene-term enrichment with a modified Fisher’s exact test. Only the pathways or biological processes with *P* < 0.05 were shown.

Given the PD-associated changes in miR-433 and miR-133b and their correlations, we performed further analyses to understand their potential target genes and similarities. Analysis in miRTarBase suggested 71 target genes for miR-433 and 67 for miR-133b (Supplementary Table S3). KEGG pathway analysis showed that the identified target genes of miR-433 were involved in 13 pathways and those of miR-133b were in 26 pathways, sharing seven pathways in common, including MAPK, PI3K-AKT, and Ras signaling pathways (**Figure 3A**). Results with statistical significance (*P* < 0.05) from GO analysis showed no shared biological processes for miR-433 and miR-133b, while the later was involved in more processes (41 vs. 6). The most significant biological processes included phosphatidylinositol-mediated signaling, positive regulation of DNA replication, regulation of actin cytoskeleton organization, and fibroblast growth factor receptor signaling pathway (**Figure 3B**).

We also applied multiple supervised learning algorithms, including decision tree, conditional inference tree, random forest, support vector machine, naive Bayesian, and logistic regression, to model miR-433 and miR-133b for PD prediction (**Table 2**). None of the models led to decent overall performance in terms of AUC, accuracy, sensitivity, and specificity based on the current data. Interestingly, a specificity of 0.87 was achieved by naive Bayesian classifier.

**Table 2:** Algorithm models of miR-433 and miR-133b for PD prediction.

Models	AUC	Accuracy	Sensitivity	Specificity
Decision tree	0.59	0.58	0.56	0.61
Conditional inference tree	0.56	0.54	0.42	0.68
Random forest	0.59	0.59	0.59	0.59
Support vector machine	0.58	0.56	0.52	0.62
Naive Bayesian	0.62	0.61	0.37	0.87
Logistic regression	0.59	0.58	0.55	0.63

*AUC, the area under the receiver operating characteristic curve.*

## Discussion

Deficiency in efficient biomarkers hinders timely intervention of PD and thus demands continuous endeavors. Aberrant miRNA expression has been suggested in a strength of linkage to sporadic PD (Harraz et al., 2011). Herein, we demonstrate that plasma levels of miR-433 and miR-133b significantly differ between PD patients and controls, following screening a set of cell-free PD-associated miRNAs. These two miRNAs exhibit strong correlations in terms of their expression levels in plasma, and share several common signaling pathways.

miR-433 binds to the promoter region of *FGF20*. A variant, rs12720208, at the miRNA binding site of *FGF20* is associated with PD, as its risk allele prevents miR-433 from attaching and leads to increased FGF20 translation and subsequent α-synuclein expression (Wang et al., 2008). The reduced expression of miR-433 as suggested in plasma may contribute to increased FGF20 and α-synuclein expression, assuming that the plasma level reflects the changes in the brain. In accordance with our results, a recent profiling of extracellular miRNAs by small RNA sequencing also suggests a significant decrease of miR-433 in cerebrospinal fluid (CSF) of postmortem PD patients. Their profiling in serum shows five differentially expressed miRNAs (**Table 3**), but no statistical difference was found in miR-433 and miR-133b (Burgos et al., 2014). One possible reason for the discrepancy is that the profiling samples are from postmortem patients, which usually are at very late stage of the disease. Meanwhile, specific analysis by qPCR may be more sensitive than general sequencing profiling.

**Table 3:** Aberrant expression of miRNAs in fluids or blood cells of PD patients.

Studies	Sources	PD, *n*	Ctrl, *n*	Method	Increased miRNA	Decreased miRNA
Margis et al., 2011	Blood	8^1^	8	qPCR		miR-1, miR-22^∗^, miR-29a
Serafin et al., 2015	Blood	36	36	qPCR	miR-29a, miR-30b, miR-103a	
Soreq et al., 2013	Leukocytes	7	6	NGS	miR-18b^∗^, miR-20a, miR-21, miR-150, miR-199b, miR-378c, miR-671, miR-1249, miR-1274b, miR-4293	miR-16, miR-92b, miR-320a, miR-320b, miR-320c, miR-769
Martins et al., 2011	PBMCs	19	13	M-array		miR-19b, miR-26a, miR-28-5p, miR-29b, miR-29c, miR-30b, miR-30c, miR-126, miR-126^∗^, miR-147, miR-151-3p, miR-151-5p, miR-199a-5p, miR-199a/b-3p, miR-301a, miR-335, miR-374a, miR-374b
Cardo et al., 2013	Plasma	31	25	qPCR/TLDA^2^	miR-331-5p	
Khoo et al., 2012	Plasma	42	30	qPCR/M-array^2^	miR-222, miR-505, miR-626	
Li et al., 2017	Plasma	60	60	qPCR	miR-137	miR-124
Current study, 2017	Plasma	46	49	qPCR		miR-133b, miR-433
Burgos et al., 2014	Serum	50	62	NGS	miR-30a-3p, miR-30e-3p, miR-338-3p	miR-16-2-3p, miR-1294
Botta-Orfila et al., 2014	Serum	65	65	TLDA		miR-19b, miR-29a, miR-29c
Cao et al., 2017	Serum	109	40	qPCR	miR-24, miR-195	miR-19b
Ding et al., 2016	Serum	106	91	qPCR/S-seq^2^	miR-195	miR-15b, miR-181a, miR-185, miR-221
Dong et al., 2016	Serum	122	104	qPCR/S-seq^2^		miR-141, miR-146b-5p, miR-193a-3p, miR-214
Ma et al., 2016	Serum	138	112	qPCR		miR-29c, miR-146a, miR-214, miR-221
Vallelunga et al., 2014	Serum	25	25	qPCR/TLDA^2^	miR-24, miR-223^∗^, miR-324-3p	miR-30c, miR-148b
Zhao et al., 2014	Serum	46	46	qPCR		miR-133b
Burgos et al., 2014	CSF	57	65	NGS	miR-19a-3p, miR-19b-3p, let-7g-3p	miR-10a-5p, miR-127-3p, miR-128, miR-132-5p, miR-136-3p, miR-212-3p, miR-370, miR-409-3p, miR-431-3p, miR-433, miR-485-5p, miR-873-3p, miR-1224-5p, miR-4448
Gui et al., 2015	CSF	78	35	qPCR/TLDA^2^	miR-10a-5p, miR-136-3p, miR-153, miR-409-3p, miR-433, let-7g-3p	miR-1, miR-19b-3p
Marques et al., 2016	CSF	28	28	qPCR	miR-205	miR-24

*CSF, cerebrospinal fluid; Ctrl, controls; M-array, microarray; NGS, next generation sequencing; PBMCs, peripheral blood mononuclear cells; PD, Parkinson’s disease; S-seq, Solexa sequencing; TLDA, TaqMan Low Density Array; ^1^Untreated PD patients; ^2^qPCR as validation after initial screening using array or sequencing.*

The miR-133b is enriched in dopaminergic neurons and is deficient in midbrain tissues of PD patients (Kim et al., 2007). The miR-133b suppresses midbrain dopaminergic neuron maturation and function by targeting Pitx3 as disclosed in primary rat midbrain cultures (Kim et al., 2007). The deficiency of miR-133b in PD is further confirmed using laser microdissected postmortem dopaminergic neurons in a case–control study (Sonntag, 2010). In accordance, miR-133b level is significantly decreased in plasma/serum of PD patients as shown in the current study and a previous report (Zhao et al., 2014). Interestingly, different from the above *in vitro* model (Kim et al., 2007), miR-133b null mice exhibit normal numbers of midbrain dopaminergic neurons during development and aging along with unchanged Pitx3 expression and dopamine release in the striatum (Heyer et al., 2012). This result indicates that miR-133b *per se* is not sufficient to induce the pathogenesis of PD, or its reduction in PD may merely be a consequence of dopaminergic neuronal loss during disease progression.

In contrast to miR-433, miR-133b, miR-34b, and miR-153, which are stably measurable in plasma, miR-34c and miR-7 are present at very low level in plasma, indicating that these two miRNAs may not be suitable to be biomarkers. On the other hand, our limited sample volume prevents us from detecting these low abundant miRNAs through the use of higher amount of RNA for the original cDNA synthesis. Results from microarray and sequencing analyses show that miR-34c and/or miR-7 are expressed in peripheral blood mononuclear cells (PBMCs) and in serum, although their expression not altered in PD (Martins et al., 2011; Burgos et al., 2014).

Pairwise correlations between miR-433, miR-133b, miR-34b, and miR-153 showed a trend of reduction in the PD group, indicating a differential involvement of these miRNAs in PD pathogenesis and/or pathology. Notably, the strongest correlation is observed between miR-433 and miR-133b, the two miRNAs with significant changes in PD. It appears that aging has no impact on levels of these miRNAs in plasma. However, further studies are needed from populations of wider age range since the age of participants in this study resides in a narrow range. It is surprising that changes in miR-433 and miR-133b expression are not associated with PD severity, although this seems not a sole case (Zhao et al., 2014; Li et al., 2017). Further studies are needed with larger sample size for each stage.

Potential target genes of miR-433 and miR-133b were involved in various pathways, sharing seven in common. However, no overlap was found in these genes, neither in the biological processes, suggesting that they function likely in a complementary manner. Modeling using data of miR-433 and miR-133b does not generate satisfactory performance for PD prediction. It may attribute to our relative small sample size that limits the cross-validation to build an optimal machine-learning model. On the other hand, it may not be surprising since a single form of biomarker has been considered almost impossible to predict PD and a combination of multiple testing including imaging, biochemical, and genetic is proposed to be required for future success (Kalia and Lang, 2015).

Proper normalization is critical for detection of authentic biological differences between samples. Although there is no consensus on the miRNA loading control (Laurent et al., 2015), many studies have used nucleolar RNA U6 as an internal control to normalize circulating miRNAs (Ai et al., 2010; Wang et al., 2013; Zhao et al., 2014), while some studies simply follow a consistent reaction system using no normalization controls as noted earlier (Chen et al., 2008; Ren et al., 2013). Besides, endogenous miR-16-5p, considered to be similar among individuals in most cases, was occasionally used as an internal control (Song et al., 2012). However, serum or plasma prepared from hemolyzed blood samples may contain miR-16-5p that can be increased by 20- to 30-fold (Pritchard et al., 2012). Exogenous synthesized miRNAs such as *Caenorhabditis elegans* miR-39/54/238 are also used for normalization (Mitchell et al., 2008; Brase et al., 2011; Tan et al., 2014), which, however, lack the capacity to normalize biological and pathological variations (Laurent et al., 2015). In general, higher amount of qPCR template can reduce the cycle number and obtain lower Ct value for potentially more stable values, and larger sample size helps the power of analysis (Dong et al., 2016). Unfortunately, there is realistic challenges to acquire more volume and more samples such as in the study by Muller et al. (2014). In the current study, some Ct values are relatively high in measurements of miR-433 and miR-153. As indicated in Section “Materials and Methods,” triplicate repeats are used during each qPCR to help the quality control of reliability. The normalization methods using U6 as an internal control and using no control lead to similar results, suggesting that our reactions are stably performed following a consistent system.

As summarized in **Table 3**, miRNA biomarkers of PD are investigated in blood, PBMCs, leukocytes, plasma, serum, and CSF. There seems no report in urine and saliva, although cases are found in other diseases such as cancer (Park et al., 2009; Yamada et al., 2011). Interestingly, the identified differentially expressed miRNAs in PD are varied among studies (**Table 3**). As mentioned earlier, we selected the six miRNAs based on postmortem evidence in brain and/or regulating α-synuclein expression. Not considering the studies which measure miRNAs individually selected by their respective rationales, results from screenings by arrays or next generation sequencing are surprisingly rarely overlapped (Martins et al., 2011; Khoo et al., 2012; Cardo et al., 2013; Soreq et al., 2013; Botta-Orfila et al., 2014; Burgos et al., 2014; Vallelunga et al., 2014; Gui et al., 2015; Ding et al., 2016; Dong et al., 2016). This discrepancy may be attributed to differences in ages, disease stages and subtypes of included patients, threshold of significance in data processing, sample sizes, and type of sources if using different fluids/cells. Of note from the overall results, a decreased level of miR-19b/miR-19b-3p in PD is suggested by four studies, respectively, in PBMCs (Martins et al., 2011), serum (Botta-Orfila et al., 2014; Cao et al., 2017), and CSF (Gui et al., 2015), except one study which shows increased miR-19b-3p expression in CSF (Burgos et al., 2014). The expression of miR-195 is suggested increased in serum of PD by two studies (Ding et al., 2016; Cao et al., 2017). The expression of miR-30c is decreased in PD, respectively, in PBMCs (Martins et al., 2011) and serum (Vallelunga et al., 2014). Decreased levels of miR-133b and miR-433 in plasma of PD as shown in the current study are in line with two previous reports, respectively in serum and CSF (Burgos et al., 2014; Zhao et al., 2014). Also, consistent with our results, miR-153 and miR-34b are not reported to be changed in fluids of PD. miR-34c and miR-7, which are not sufficiently detected in our condition, are not suggested to be changed either in PD (**Table 3**). Further investigations are warranted to clarify how levels of miRNAs change in the fluids of PD in association with PD subtypes and disease progression. Also, the differences may be worth testing in urine and saliva in order to further understand their potentiality to serve as PD biomarkers.

## Conclusion

While it is a long haul to eventually identify clinic proven biomarkers, we herein screen and demonstrate that levels of miR-433 and miR-133b, two miRNAs associated with PD pathogenesis, are significantly reduced in plasma of PD patients and not correlated with age, suggesting their specificity and potentiality to serve as biomarkers. Future studies are needed in combination with other testing to upgrade their performance in predicting PD.

## Author Contributions

J-HZ, XZ, and H-MW designed and supervised the project. XZ, B-LH, and Z-YH collected samples and clinical data. RY and L-LZ performed research. RY, PL, and H-MW analyzed data. J-HZ and RY wrote the article.

## Conflict of Interest Statement

The authors declare that the research was conducted in the absence of any commercial or financial relationships that could be construed as a potential conflict of interest.

## Footnotes

1 mirtarbase.mbc.nctu.edu.tw

2 david.abcc.ncifcrf.gov
