# Supplementary material for: Reduced Circulating Levels of miR-433 and miR-133b Are Potential Biomarkers for Parkinson’s Disease
Source: Front Cell Neurosci. 2017 Jun 23;11:170. doi: 10.3389/fncel.2017.00170 (PMC5481393; doi:10.3389/fncel.2017.00170)
Supplement: Supplementary file 1 [file Presentation_1.pdf]

Supplementary Material:

Reduced Circulating Levels of miR-433 and miR-133b Are Potential Biomarkers for Parkinson's Disease

Xiong Zhang[1,2], Rui Yang[2], Bei-Lei Hu[1], Pengcheng Lu[3], Li-Li Zhou[2], Zhi-Yong He[1], Hong-Mei Wu[2]*, Jian-Hong Zhu[1,2,4]*

* Correspondence:
Jian-Hong Zhu:
jhzhu@wmu.edu.cn
Hong-Mei Wu:
hmwu@wmu.edu.cn

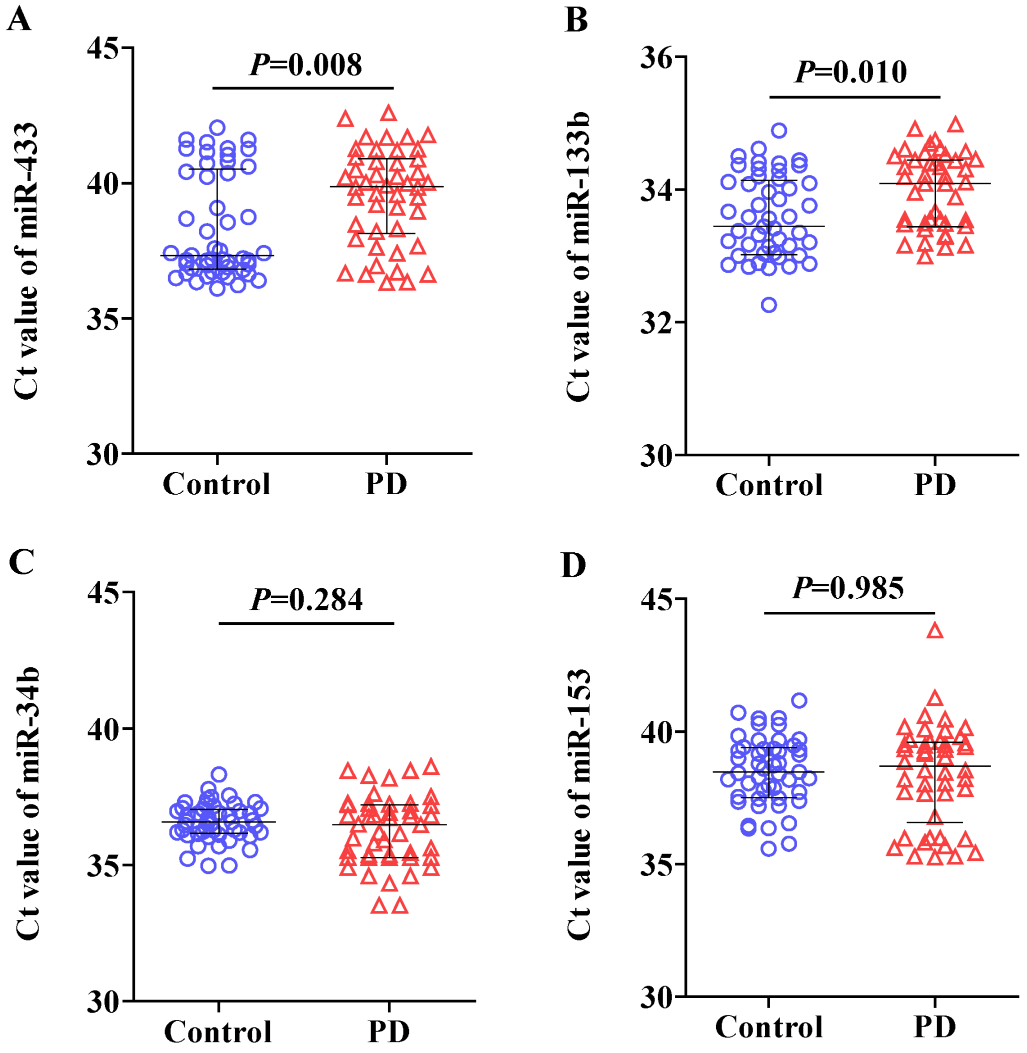

Figure S1. Ct values of miR-433 (**A**), miR-133b (**B**), miR-34b (**C**) and miR-153 (**D**) qPCR in plasma of the Parkinson's disease (PD) patients and controls. The bar represents median with interquartile range.

Table S1. Correlation analyses between age and expression levels of miR-433, miR-133b, miR-34b and miR-153 in the control and PD groups

	Age (control)			Age (PD)		
	r_s	95% CI	*P*	r_s	95% CI	*P*
miR-433	0.02	-0.29~0.34	0.91	0.09	-0.20~0.35	0.55
miR-133b	-0.06	-0.35~0.26	0.70	0.09	-0.21~0.39	0.58
miR-34b	0.07	-0.22~0.37	0.62	0.12	-0.19~0.43	0.44
miR-153	0.21	-0.13~0.53	0.15	0.10	-0.22~0.41	0.53

PD, Parkinson's disease; r_s, Spearman's correlation coefficient; CI, confidence interval.

Table S2. Association analyses of miRNA expression levels with disease severity

Subjects	Stage I (N=20)	Stage II (N=11)	Stage III (N=12)	*P*
Age (years); mean ±SEM	61.55 ±2.63	60.18 ±2.45	67.40 ±1.81	0.12
Gender	M: 7 (35%)	M: 6 (55%)	M: 7 (58%)	0.84
	F: 13 (65%)	F: 5 (45%)	F: 5 (42%)	
miR-433; median (range)	0.02 (0.003-0.45)	0.01 (0.002-0.09)	0.01 (0.002-0.25)	0.18
miR-133b; median (range)	0.92 (0.26-4.76)	0.66 (0.05-1.65)	0.44 (0.06-7.75)	0.38
miR-34b; median (range)	0.16 (0.05-2.12)	0.12 (0.01-1.08)	0.12 (0.01-1.35)	0.74
miR-153; median (range)	0.07 (0.003-0.54)	0.06 (0.002-0.77)	0.04 (0.001-0.60)	0.44

Difference in age and gender was analyzed using ANOVA and χ^2 test, respectively; Difference in miRNA expression levels among the three stages was analyzed using Kruskal-Wallis test.

Table S3. The target genes of miR-433 and miR-133b

**miR-433 target genes**		**miR-133b target genes**	
Gene ID	Official full name	Gene ID	Official full name
ALDOA	aldolase A, fructose-bisphosphate	AKT1	v-akt murine thymoma viral oncogene homolog 1
ATXN1	ataxin 1	ANGPT4	angiopoietin 4
AZIN1	antizyme inhibitor 1	ATP13A3	ATPase 13A3
BBS2	Bardet-Biedl syndrome 2	BCL2L2	BCL2-like 2
BRWD1	bromodomain and WD repeat domain containing 1	C11orf24	chromosome 11 open reading frame 24
C5ORF22	chromosome 5 open reading frame 22	C17orf64	chromosome 17 open reading frame 64
CFTR	cystic fibrosis transmembrane conductance regulator (ATP-binding cassette sub-family C, member 7)	CASP9	caspase 9
CHD9	chromodomain helicase DNA binding protein 9	CCDC39	coiled-coil domain containing 39
COX6B1	cytochrome c oxidase subunit Vib polypeptide 1 (ubiquitous)	CCNI	cyclin I
DGKE	diacylglycerol kinase, epsilon 64kDa	CDC42	cell division cycle 42
EDN1	endothelin 1	CDK13	cyclin-dependent kinase 13
ENTPD4	ectonucleoside triphosphate diphosphohydrolase 4	CDK5R1	cyclin-dependent kinase 5, regulatory subunit 1 (p35)
ERBB2IP	erbb2 interacting protein	CHMP3	charged multivesicular body protein 3
FAM126B	family with sequence similarity 126, member B	CMTM4	CKLF like MARVEL transmembrane domain containing 4
FAM3C	family with sequence similarity 3, member C	CNN2	calponin 2
FGF20	fibroblast growth factor 20	CPNE3	copine 3
FGF9	fibroblast growth factor 9 (glia-activating factor)	CTGF	connective tissue growth factor
FKBP1A	FK506 binding protein 1A, 12kDa	CXCR4	chemokine (C-X-C motif) receptor 4

FKBP1C	FK506 binding protein 1C
GABPB1	GA binding protein transcription factor, beta subunit 1
GBP2	guanylate binding protein 2, interferon-inducible
GPR135	G protein-coupled receptor 135
GRB2	growth factor receptor-bound protein 2
HAUS2	HAUS augmin-like complex, subunit 2
HDAC6	histone deacetylase 6
HIVEP1	human immunodeficiency virus type I enhancer binding protein 1
HRH4	histamine receptor H4
HSP90B1	heat shock protein 90kDa beta (Grp94), member 1
ITIH5	inter-alpha (globulin) inhibitor H5
KLHL4	kelch-like 4 (Drosophila)
KRAS	v-Ki-ras2 Kirsten rat sarcoma viral oncogene homolog
LEMD2	LEM domain containing 2
LONRF2	LON peptidase N-terminal domain and ring finger 2
LONRF3	LON peptidase N-terminal domain and ring finger 3
LRIG3	leucine-rich repeats and immunoglobulin-like domains 3
MAFK	v-maf musculoaponeurotic fibrosarcoma oncogene homolog K (avian)
MAPK8	mitogen-activated protein kinase 8
MBNL1	muscleblind-like (Drosophila)
DCAKD	dephospho-CoA kinase domain containing
DUX4L9	double homeobox 4 like 9
EGFR	epidermal growth factor receptor
EMID1	EMI domain containing 1
ERG	v-ets avian erythroblastosis virus E26 oncogene homolog
FAIM	Fas apoptotic inhibitory molecule
FAM160B1	family with sequence similarity 160 member B1
FGFR1	fibroblast growth factor receptor 1
FOSL2	FOS like antigen 2
FOXL2	forkhead box L2
FSCN1	fascin actin-bundling protein 1
GLI1	GLI family zinc finger 1
HCN2	hyperpolarization activated cyclic nucleotide gated potassium channel 2
HCN4	hyperpolarization activated cyclic nucleotide gated potassium channel 4
HIST2H2AC	histone cluster 2, H2ac
IGF1R	insulin like growth factor 1 receptor
ITPKB	inositol-trisphosphate 3-kinase B
KCNH2	potassium voltage-gated channel subfamily H member 2
KLF15	Kruppel-like factor 15
MC2R	melanocortin 2 receptor

MRPS25	mitochondrial ribosomal protein S25	MCL1	myeloid cell leukemia 1
MTMR9	myotubularin related protein 9	MET	MET proto-oncogene, receptor tyrosine kinase
NEK9	NIMA (never in mitosis gene a)- related kinase 9	MMP9	matrix metallopeptidase 9
NINJ1	ninjurin 1	MYPN	myopalladin
NSA2	hypothetical gene supported by NM_014886; TGF beta-inducible nuclear protein 1; similar to TGF beta-inducible nuclear protein 1	PDE1A	phosphodiesterase 1A
NUBPL	nucleotide binding protein-like	PITX3	paired like homeodomain 3
PDLIM3	PDZ and LIM domain 3	PKM	pyruvate kinase, muscle
PLEKHA1	pleckstrin homology domain containing, family A (phosphoinositide binding specific) member 1	PRDM16	PR domain 16
PTPLAD1	protein tyrosine phosphatase-like A domain containing 1	PTBP2	polypyrimidine tract binding protein 2
RPL24	ribosomal protein L24; ribosomal protein L24 pseudogene 6	PTMA	prothymosin, alpha
RUNX2	runt-related transcription factor 2	PTPRK	protein tyrosine phosphatase, receptor type K
SCAMP1	secretory carrier membrane protein 1	RB1CC1	RB1 inducible coiled-coil 1
SCNN1G	sodium channel, nonvoltage-gated 1, gamma	RBMXL1	RNA binding motif protein, X-linked-like 1
SERBP1	SERPINE1 mRNA binding protein 1	RHOA	ras homolog family member A
SLC1A5	solute carrier family 1 (neutral amino acid transporter), member 5	RHOQ	ras homolog family member Q
SLC28A1	solute carrier family 28 (sodium-coupled nucleoside transporter), member 1	RNF103-CHMP3	RNF103-CHMP3 readthrough
SMU1	smu-1 suppressor of mec-8 and unc-52 homolog (C. elegans)	RNF168	ring finger protein 168, E3 ubiquitin protein ligase

SPATS2L	spermatogenesis associated, serine-rich 2-like	SERPINH1	serpin peptidase inhibitor, clade H (heat shock protein 47), member 1, (collagen binding protein 1)
SPRYD4	SPRY domain containing 4	SESN3	sestrin 3
SSC5D	hypothetical LOC284297	SP1	Sp1 transcription factor
STARD7	StAR-related lipid transfer (START) domain containing 7	STK3	serine/threonine kinase 3
STK38	serine/threonine kinase 38	SUPT16H	SPT16 homolog, facilitates chromatin remodeling subunit
TCF20	transcription factor 20 (AR1)	SYAP1	synapse associated protein 1
TFAP2A	transcription factor AP-2 alpha	TAGLN2	transgelin 2
TGIF2LX	TGFB-induced factor homeobox 2-like, X-linked	TRIM71	tripartite motif containing 71, E3 ubiquitin protein ligase
TM4SF5	transmembrane 4 L six family member 5	TXNRD3NB	thioredoxin reductase 3 neighbor
TMEM229B	chromosome 14 open reading frame 83	ZBTB37	zinc finger and BTB domain containing 37
TYMS	thymidylate synthetase	ZFP28	ZFP28 zinc finger protein
UGT2B4	UDP glucuronosyltransferase 2 family, polypeptide B4	ZMAT4	zinc finger matrin-type 4
WDR45B	WD repeat domain 45B		
YIPF6	Yip1 domain family, member 6		
ZNF584	zinc finger protein 584		
ZNF780A	zinc finger protein 780A		
